# Evaluation of the risk of lymphomagenesis in xenografts by the PCR-based detection of EBV BamHI W region in patient cancer specimens

**DOI:** 10.18632/oncotarget.10322

**Published:** 2016-06-29

**Authors:** Junko Mukohyama, Dai Iwakiri, Yoh Zen, Toru Mukohara, Hironobu Minami, Yoshihiro Kakeji, Yohei Shimono

**Affiliations:** ^1^ Division of Molecular and Cellular Biology, Kobe University Graduate School of Medicine, Kobe, Japan; ^2^ Division of Gastrointestinal Surgery, Kobe University Graduate School of Medicine, Kobe, Japan; ^3^ Division of Clinical Virology, Kobe University Graduate School of Medicine, Kobe, Japan; ^4^ Department of Diagnostic Pathology, Kobe University Graduate School of Medicine, Kobe, Japan; ^5^ Division of Medical Oncology/Hematology, Kobe University Graduate School of Medicine, Kobe, Japan; ^6^ Cancer Center, Kobe University Hospital, Kobe, Japan

**Keywords:** patient-derived tumor xenograft, lymphomagenesis, Epstein-Barr virus, colorectal cancer, BamHI W region

## Abstract

Establishment of patient-derived tumor xenografts (PDXs) is hampered by lymphomagenesis mostly caused by the latently-infected Epstein-Barr virus (EBV) contained in patient cancer tissues. However, the character of patient tissues that result in lymphomagenesis after xenotransplantation is not elucidated. In this study, we analyzed the patient colorectal cancer (CRC) tissues and the PDXs established by their xenotransplantation. We found that 2 of 9 (22%) PDX tumors were EBV-associated human diffuse large B cell lymphoma which was formed by clonal proliferation of human B-cell lymphocytes, were strongly positive for EBER-ISH, and were classified as type III latency. Expression of EBV genes and RNAs, such as EBNAs, LMP1, EBER and EBV-associated microRNAs in patient CRC tissues were unlikely to be associated with lymphomagenesis in PDXs. In contrast, the positive PCR-based amplification of BamHI W region, a major internal repeat in EBV genome, in the patient CRC tissues was correlated with lymphomagenesis in PDXs. These results suggest that the detection of the EBV BamHI W region in the patient surgical specimens will be an effective way to predict the risk of lymphomagenesis in PDXs before xenotransplantation.

## INTRODUCTION

Patient-derived tumor xenografts (PDXs) are established by xenotransplantation of surgically resected human cancer specimens into immunodeficient mice, such as a nonobese diabetic/severe combined immunodeficientcy (NOD/SCID) mouse which is deficient in both innate and adaptive immunities, and an NOD/SCID/IL-2rgnull (NSG) mouse which further lacks NK cell activity. PDX tumors are able to mirror patients' histopathological and genetic profiles, can metastasize to distant organs, and are characterized by the presence of a cancer stem cell (CSC) population [1-3]. Furthermore, PDXs are an attractive preclinical model to investigate therapeutic response, cancer resistance, metastasis and biomarkers [4, 5].

It is reported that the engraftment of human cancer specimens into an immunodeficient mouse unexpectedly resulted in the formation of lymphoma instead of their corresponding cancers, such as lung, liver, gastric, bladder, breast and prostate cancers [6-10]. The incidence of lymphomagenesis among the established PDXs differ widely depending on the cancer types; for example, 1/30 PDXs (3.3 %) in colorectal cancer (CRC), 11/21 (56%) PDXs derived from 16 patients in liver cancer, and 8/10 PDXs (80%) in prostate cancer [7, 8, 10]. In addition, transplantation of the same cancer specimen resulted in the formation of a lymphoma PDX in one mouse and a cancer PDX in another mouse [9]. Pathological analyses of 26 lymphoma PDXs revealed that 23 PDXs were Epstein-Barr virus (EBV)-associated human B cell lymphoma, and the remaining 3 PDXs were lymphoma of mouse origin [8].

EBV is a member of human-restricted herpes virus. While EBV is well known as the causative agent of infectious mononucleosis, it is associated with various malignancies, such as Burkitt's lymphoma, Hodgkin's lymphoma, nasopharyngeal cancer, gastric cancer, and post-transplant lymphoproliferative disorders (PTLDs) [11]. PTLDs express 12 EBV latent gene products, including 6 EBV nuclear antigens (EBNA1, 2, 3A-C and leader protein), 3 latent membrane proteins (LMP1, 2A and 2B), BamHI-A rightward transcripts (BARTs) and 2 EBV-encoded small RNAs (EBER1 and 2) [11]. EBV efficiently infects resting B cells *in vitro* and transforms them into indefinitely proliferating lymphoblastoid cell lines (LCLs). Because LCLs express a similar viral gene profile, it is considered as a model to study PTLDs and immune responses [12, 13].

EBV infects more than 90% of humans and produce latent infection for the life of host. However, persistent infection of EBV is basically benign in healthy carriers because EBV reactivation and subsequent proliferation of EBV-infected lymphocytes are suppressed by immunosurveillance. In contrast, in immunodeficient individuals, EBV can transform B cell into a proliferative state and eventually cause PTLDs [14, 15]. It is considered that proliferation of the EBV-infected lymphocytes contained in the human cancer tissues occurs after xenotransplantation because of the insufficient immunosurveillance in the immunodeficient mice [6-10].

Lymphomagenesis after the xenotransplantation of human cancer tissues results in the loss of patient samples and waste of immunodeficient mice. However, it is still difficult to predict lymphomagenesis before xenotransplantation. We studied the pathological and molecular characteristics of surgically resected human CRC tissues, as well as both CRC PDXs and EBV-associated human lymphoma formed by their xenotransplantation. Our results suggest that the amount and/or presence of EBV itself in the cancer tissues for xenotransplantation are one of the factors that are associated with lymphomagenesis in the human cancer PDXs.

## RESULTS

### Lymphomagenesis by the xenotransplantation of human CRC tissues

To establish human CRC PDXs, we engrafted 13 fresh surgical specimens of CRC patients subcutaneously into the NOD/SCID or NSG mice and 9 PDXs were established. The clinicopathological features of the patients and the PDXs are presented in Table 1.

**Table 1:** Characteristics of PDX tumors and their corresponding CRC patients

	PDX histology	Pathology	Preoperative chemotherapy	TNM^a^	Mouse strain	Days for growth^b^
KUC1	CRC^c^	mod.^c^	None	pT3 pN1 cM1	NOD/SCID	75
KUC2	CRC	mod.	None	pT4a pN0 cM1	NOD/SCID	30
KUC3	No tumor	mod.	None	pT4a pN2 cM0	NOD/SCID	-
KUC4	DLBCL^c^	mod.	None^d^	pT2 pN0 cM0	NOD/SCID	28
KUC5	CRC	mod.	None	pT3 pN0 cM1	NOD/SCID	176
KUC6	CRC	mod.	None	pT3 pN1 cM0	NSG	223
KUC7	CRC	mod.	None	pT2 pN0 cM0	NSG	35
KUC8	CRC	mod.	None	pT4a pN1 cM1	NSG	47
KUC9	CRC	mod.	None	pT4b pN0 cM0	NSG	19
KUC10	No tumor	mod.	None	pT3 pN0 cM0	NSG	-
KUC11	DLBCL	well.^c^	None	pT3 pN1 cM0	NSG	55
KUC12	No tumor	well.	None	pT3 pN1 cM1	NSG	-
KUC13	No tumor	mod.	Yes	pT3 pN2 cM1	NSG	-

a TNM classification (7th edition) in clinical pathological reports.

b Number of days when PDX tumor reached 0.5 cm^3^ after xenotransplantation of patient specimen.

c Abbreviations: CRC, colorectal cancer; DLBCL, diffuse large B cell lymphoma; mod, moderately-differentiated adenocarcinoma; well, well-differentiated adenocarcinoma.

d None, but under an immunosuppressive therapy after organ transplantation.

**Figure 1:**
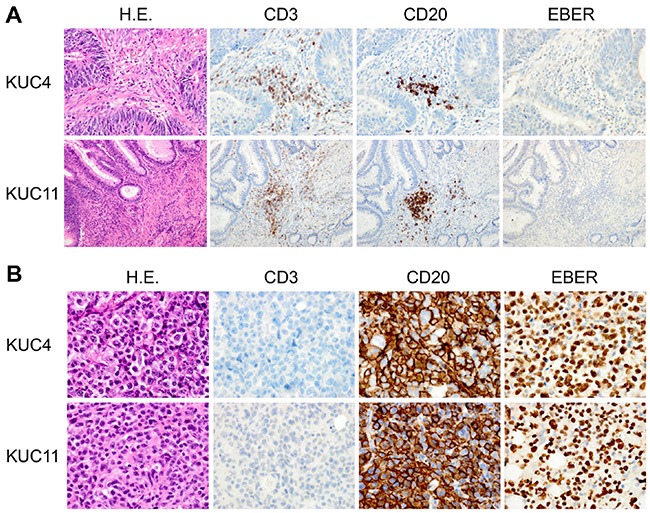
Establishment of the DLBCL PDXs from human CRC specimens **A.** Representative sections of the patient CRC samples that gave rise to the DLBCL PDXs. H.E. (left panels), immunohistochemistry for CD3 and CD20 (middle panels), and EBER ISH (right panels) are shown (original magnification, ×200 for KUC4, x100 for KUC11). Immunohistochemical analyses showed the infiltration of CD3-positive or CD20-positive lymphocytes in the tumor (brown, middle panels); but EBER was undetectable by ISH. **B.** Representative sections of the established DLBCL PDXs. H.E. (left panels), immunohistochemistry for CD3 and CD20 (middle panels), and EBER ISH (right panels) are shown (original magnification, ×400). The cells in the PDXs were exclusively stained with an anti-CD20 antibody (brown). The cells were also strongly positive for EBER ISH (brown).

**Figure 2:**
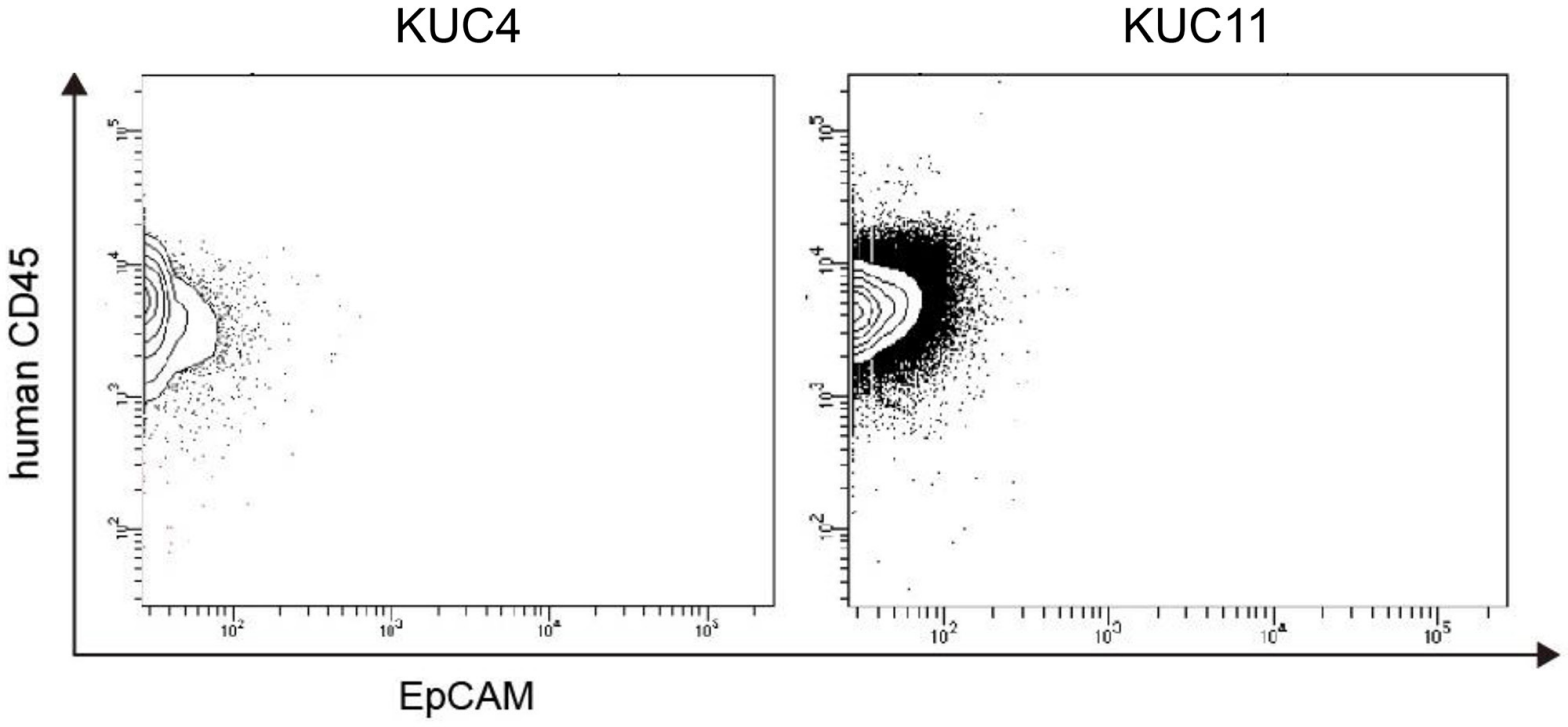
Flow cytometry analysis of the DLBCL PDXs Representative flow cytometry profiles of the isolated cells from the DLBCL PDXs (KUC4 and KUC11). Most of the PDX tumor cells were within a human CD45^+^/EpCAM^−^ population, which corresponds to human lymphocytes.

Histological examination showed that 7 of 9 (78%) PDX tumors recapitulated histopathological characteristics of the patient CRC tissues (Supplementary Figure S1). But 2 PDXs (KUC4 and KUC11) did not resemble the parent CRC tissues but exhibited the morphological characteristics of lymphoma (Figure 1). Hematoxylin and eosin (H.E.) staining showed that both lymphoma tissues were composed of large atypical lymphoid cells arranged in a diffuse fashion, against the background of scattered small non-dysplastic lymphocytes. On immunostaining and *in situ* hybridization, the neoplastic cells were diffusely positive for a B cell marker CD20 and EBER, but negative for a T cell marker CD3 (Figure 1B). These morphological features were consistent with diffuse large B-cell lymphoma (DLBCL). Histology review of the patient CRC tissues revealed occasional small lymphoid aggregates consisting of mixed CD3-positive T lymphocytes and CD20-positive B lymphocytes, but neither dysplastic lymphocytes nor EBER-positive cells were found (Figure 1A). Flow cytometric analyses of KUC4 and KUC11 DLBCL PDXs showed that tumor cells within the PDXs were predominantly positive for human CD45 and negative for human EpCAM (Figure 2), confirming that these tumors originated from human lymphocytes presented in the surgical specimens.

**Figure 3:**
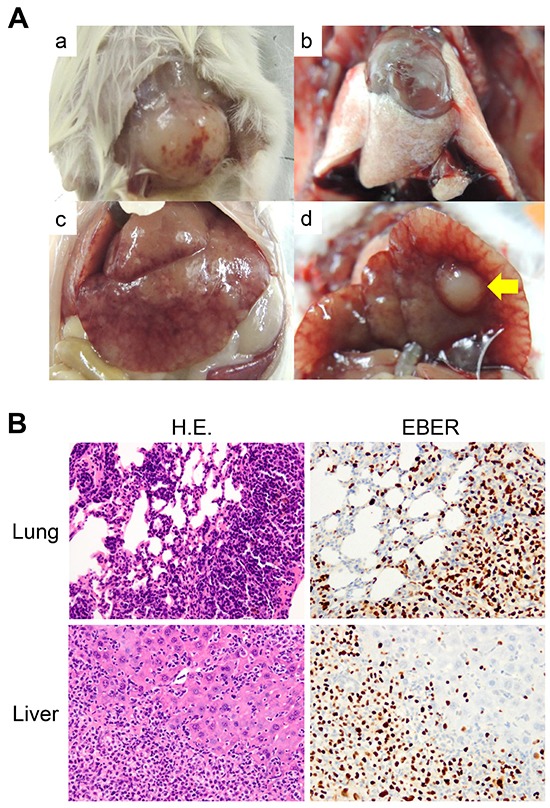
Formation of the DLBCL PDX and its distant metastases **A.** Macroscopic views of the DLBCL PDX tumor and its metastatic organs (a. DLBCL PDX tumor, b. lung, c and d. liver). No tumor mass was grossly apparent in the lungs, while a large nodular metastasis was observed in the liver (yellow arrow). **B.** Diffuse infiltration of lymphoma cells in the lung and liver. H.E. (left panels) and EBER ISH (right panels) are shown (original magnification, ×100 for the lung, x200 for the liver). The infiltrated lymphocytes were strongly positive for EBER ISH (brown).

DLBCL caused by proliferation of EBV-infected lymphocytes in human immunocompromised hosts metastasizes to distant organs. And DLBCL PDXs tend to form large metastases and grow faster than CRC PDXs which basically do not metastasize when grown subcutaneously in immunocompromised mice [9, 16]. As shown in Table 1, the days for growth tended to be shorter in DLBCL PDX than in CRC PDX. We examined the distant organs of the KUC4 and KUC11 and found metastatic lymphoma in the lungs and liver of KUC11 mouse (Figure 3A), but not in KUC4 mouse. Histologically, the metastatic tumors consisted of diffusely arranged large lymphoid cells positive for EBER, the appearance nearly identical to the subcutaneous tumor. The tumor cells widely invaded into the adjacent liver and pulmonary parenchyma (Figure 3B).

### Clonal expansion of EBV-infected lymphocytes

Because latent infection of EBV is most commonly observed in humans, we evaluated the presence of EBV by ISH for EBER. We found that both KUC4 and KUC11 were strongly positive for EBER (Figure 1B). In contrast, EBER ISH was negative for the lymphocytes within the patient specimens from which the DLBCL PDXs derived, suggesting that the amount of EBV was extremely low and below the detection threshold for EBER ISH (Figure 1A). We then analyzed the expression of EBNAs and LMP1 in the patient specimens and the DLBCL PDXs. We found that EBNA1, EBNA2, EBNA3C, and LMP1 were detectable in both DLBCL PDXs (Supplementary Figure S2), suggesting these lymphoma mimic DLBCL with type III latency. In the patient specimen of KUC11, EBNA 1, EBNA2, EBNA3C, and LMP1 were undetectable (Supplementary Figure S2). In contrast, the expression of EBNA 1, EBNA2, EBNA3C and LMP1 was detectable in the patient specimen of KUC4 (Supplementary Figure S2). We speculate that the upregulation of LMP1, a major transforming protein of EBV, in the patient specimen of KUC4 affected the much faster growth of KUC4 DLBCL PDX than that of KUC11(Table 1, 28 days vs 55 days).

**Figure 4:**
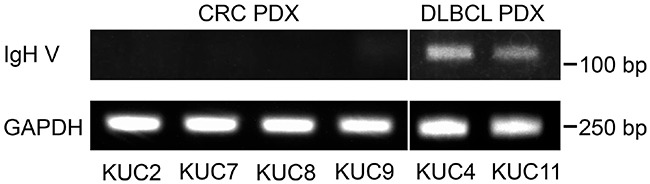
IgH gene rearrangements in the DLBCL PDXs Presence of the dominant rearrangements of IgH gene in the PDXs was analyzed with the PCR amplification of the human IgH variable (IgH V) region. Unique dominant rearrangements that correspond to clonal B-cell proliferation were detectable in the DLBCL PDXs, but not in the CRC PDXs. GAPDH was amplified as an internal control.

Then, we performed the clonality analyses of the lymphocytes by immunoglobulin heavy chain (IgH) gene rearrangement assay. The unique dominant rearrangements that correspond to clonal B-cell proliferation, were detectable in the DLBCL PDXs, but not in the CRC PDXs (Figure 4), consistent with the notion that DLBCL is induced by the clonal expansion of the human B lymphocytes.

### PCR-based detection of EBV BamHI W region in patient cancer specimens correlates with lymphomagenesis

EBV latent infection is highly prevalent among humans and relatively high lymphocyte infiltration are commonly observed in cancer tissues of human CRC specimens. Because the formation of DLBCL PDX by proliferation of EBV-infected lymphocytes results in the failure of the establishment of CRC PDXs, we examined the molecular factors that will be associated with the emergence of DLBCL PDXs.

The frequency of EBV-infected cells in peripheral blood of normal donors ranges approximately 1 in 10,000 to 1 in 100,000 memory B cells [17]. In the salivary gland, it is shown that there is less than 1 copy of EBV DNA per 1 x10^5^ cells [18]. Thus, we hypothesized that the difference of the amount of EBV in the surgical specimens affects the incidence of lymphomagenesis in the PDXs.

**Figure 5:**
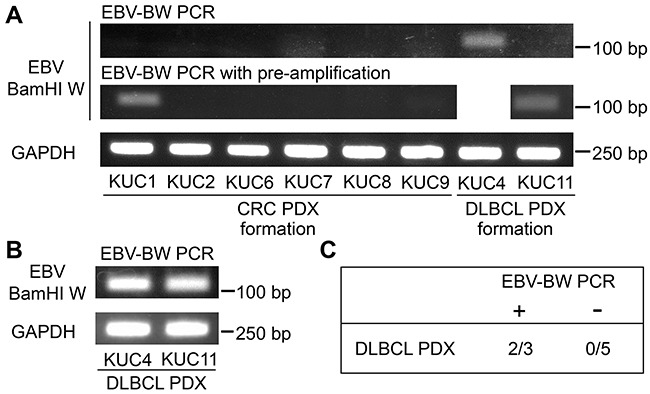
Amplification of EBV BamHI W region in the patient CRC specimens **A.** RT-PCR analyses of the EBV BamHI W region in the patient CRC specimens which gave rise to the CRC or DLBCL PDXs. The amplification of BamHI W region was detected in 1 of 8 patient CRC specimens (KUC4) by EBV-BW PCR, and became detectable in 2 of 7 patient CRC specimens (KUC1 and KUC11) after the pre-amplification of templates (p-EBV-BW PCR). GAPDH was amplified as an internal control. **B.** RT-PCR analyses of the EBV BamHI W region in the DLBCL PDXs. BamHI W region was amplified by EBV-BW PCR. GAPDH was amplified as an internal control. **C.** Incidence of lymphomagenesis by the xenotransplantation of the patient CRC specimens positive or negative for EBV-BW PCR. Lymphomagenesis was observed in 2 of 3 EBV-BW PCR/p-EBV-BW PCR positive CRC specimens.

First, we analyzed the presence of EBV BamHI W region by PCR (EBV-BW PCR) in the patient specimens. Detection of BamHI W region, a major internal repeat in EBV, is one of the most established methods to prove the presence of EBV genome [19]. We extracted 60 ng genomic DNA from the surgical specimens of the CRC patients, except for that of KUC5 which we obtained was too small to be stored. EBV BamHI W region was amplified by EBV-BW PCR in KUC4, but not in other 7 patient specimens analyzed (Figure 5A, upper panel). When the DNA samples were pre-amplified by 15 cycles and used as templates (p-EBV-BW PCR), we observed the amplification of EBV BamHI W region in 2 out of 7 patient specimens (KUC1 and KUC11, Figure 5A, middle panels). We confirmed that much higher amplification of EBV BamHI W region was observed using the DLBCL PDXs, KUC4 and KUC11, as templates (Figure 5B). When the EBV BamHI W region was detectable by EBV-BW PCR or p-EBV-BW PCR, the transplantation of the samples resulted in lymphomagenesis in 2 out of 3 patient cancer specimens (Figure 5C). In contrast, when the EBV BamHI W region was undetectable by EBV-BW PCR or p-EBV-BW PCR, no patient cancer specimens resulted in lymphomagenesis (0 out of 7 patient cancer specimens). These result suggest that the PCR-detection of EBV in the patient CRC tissue is correlated with lymphomagenesis in the PDX.

Because the EBV-associated microRNAs are expressed in EBV-infected cells, we also analyzed the expression of BART 1-5p and BART 7 by RT-PCR. The expression levels of these miRNAs were extremely high in the DLBCL PDXs (Supplementary Figure S3), but were low or hardly detectable in the patient CRC specimen of KUC11 in which the amplification of the EBV BamHI W region was weak (Figure 5, Supplementary Figure S3). In contrast, these miRNAs were detectable in the patient specimen of KUC4 in which higher amplification of the EBV BamHI W region and the expression of EBNA genes were observed (Figure 5A, Supplementary Figures S2A and S3).

Taken together, these results suggest that the detection of EBV BamHI W region from the CRC surgical specimens with p-EBV-BW PCR will be an effective way to evaluate the risk of lymphomagenesis before xenotransplantation.

## DISCUSSION

PDX model is an attractive model to analyze the character of cancer cells within the patient cancer tissues. However, establishment of PDX is occasionally hampered by the lymphomagenesis, mostly caused by proliferation of EBV-infected lymphocytes. Lymphomagenesis in the PDX is reported in other types of cancers, such as lung, liver, gastric, bladder, breast, prostate, and colorectal cancers [6-10]. Most of them exhibit histological character of EBV-associated DLBCL. In this study, we analyzed 13 human CRC tissues that resulted in the formation of 2 lymphomas and 7 histologically confirmed CRC PDXs. It has been reported that the tumor take rate of human primary CRC tissue in immunodecifient mice ranges between 63.5-84.4% which is relatively higher than other types of cancers, such as breast and prostate cancers [1, 20]. Thus, the rate of establishment of CRC PDX (53.8% (7/13)) and any PDXs including CRC and DLBCL PDXs (69.2% (9/13)) in this study is close to or within the range of the previous reports. In this study, 2 out of 9 PDXs showed morphological characteristics of lymphoma (22.2%). Although the number of the reports on the lymphomagenesis in PDXs are still limited, other two reports present that the incidence of lymphomagenesis in the PDX transplanted with human CRC tissues was 3.3% (1/30) and 28.6% (2/7) [8, 9], suggesting that it is important to consider a possibilities of the lymphomagenesis when establishing PDX using the patient CRC tissues.

Several factors are reported to be associated with lymphomagenesis in the PDXs: low tumorigenicity of cancer cells in the patient primary tissues, preoperative chemotherapy, and inflammation in the tissues. In contrast, the factors, such as patient stage and vascular invasion, are not associated with the rate of lymphomagenesis. For example, the frequency of lymphomagenesis is up to 80% in the prostate cancer PDX which will be attributed to the low tumorigenicity of the androgen-sensitive prostate cancer [10]. Zhang et al. suggested that cancer type, preoperative chemotherapy and inflammation in patient primary cancer were associated with high risk of lymphomagenesis in PDX [8]. They suggested that gastric cancer specimens are more likely form DLBCL PDX than CRC ones, because of chronic mucosal inflammation caused by Helicobacter pylori. In this study which focused on CRC, We found that KUC1 did not result in the formation of DLBCL lymphoma, even though the specimen was positive for p-EBV-BW PCR. We speculate that KUC1 specimen obtained from the patient of the metastatic CRC was more tumroigenic than other non-metastatic cancer specimens, such as KUC4 and KUC11 (Table 1) and that lymphomagenesis was competitively suppressed by the engraftment and proliferation of CRC cells in the patient specimen.

The mechanism of lymphomagenesis in immunodeficient mice transplanted with human cancer is similar to that occur in severe immunodeficient patients, such as acquired immune deficiency syndrome and post-transplanted patients. Lymphoproliferative disorders in human immunodeficient patients are caused by the pathogens, such as EBV, HSV8, and HTLV-1. Among them, EBV is the dominant cause of lymphomagenesis in the PDXs reported to date [6-10]. EBV-associated lymphoma can be classified into the three latency types based on the expression of the latent EBV genes [21]. Briefly, type I, type II and type III latency are characterized by the expression of EBNA1, EBNA1 and LMP1, and EBNA 1, 2, 3 and LMP1, respectively. The expression profile of EBNA 1-3 and LMP1 confirmed that both of our DLBCL PDXs (KUC4 and KUC11) were latency type III lymphoma, which is observed in the patients under severe immunodeficient state. Consistent with our observation, the histology of other reported lymphomas is DLBCL. These results strongly suggest that the lymphoma PDXs are formed by proliferation of EBV-infected lymphocytes existed in the transplanted human cancer tissues. In this study, we analyzed the expression of BART miRNAs because BART miRNAs are reported as major contributors to the transformed growth properties of the EBV-infected cells [22]. The BART miRNAs can be detected by PCR in all forms of EBV latency, and their highest expression is detected in latency type I and II [23, 24]. We analyzed the expression level of the BART miRNA, such as BART 1-5p and BART7, in DLBCL PDX and corresponding patient CRC tissue. Consistent with their roles in transformation of lymphocytes, the expression levels of these miRNA were extremely high in the DLBCL PDXs (Supplementary Figure S3).

Although the expression of EBNAs and BART miRNAs were observed in the patient specimen of KUC4, this patient who received immunosuppressive therapies presented no clinical and pathological signs of lymphoma (Figure 1A, Table 1 Supplementary Figures S2 and S3). It is possible that proliferation of the EBV-infected lymphocytes was not induced in this patient at least partly because the expression of LMP1, an EBV genes required for transformation [25], was weak and was 10^−3^ times as much as that of the KUC4 DLBCL PDX (Supplementary Figure S2). Considering that the transplantation of KUC4 CRC specimen resulted in DLBCLPDX, it is possible that upregulation of the EBV genes in patient primary tissue is one of the factors that affect the lymphomagenesis in PDXs. On the other hand, because EBNA and LMP1 mRNAs and miRNAs were undetectable or very weakly expressed in all other patient specimens (Supplementary Figures S2 and S3), we could not correlate upregulation of the EBV genes and miRNAs in the patient specimens with the lymphomagenesis. Taken together, our results suggest that upregulation of EBV genes and miRNAs in patient tissue may not be associated with lymphomagenesis unless the patient is under severe immunosuppressive state.

In healthy EBV carriers, latent infection of EBV is basically limited to memory B cells that are in a resting state and no viral proteins are produced from the genome [26-28]. Because memory B cells exist in lymph nodes and rarely exist in blood and tissues, amount of EBV virus particles in the patient tissues may differ depending on the patient samples. In this study, we first applied EBV-BW PCR, to detect the presence of EBV in the patient cancer tissues for xenotransplantation. The presence of EBV is hardly detectable in the patient cancer tissues, except for KUC4, which was derived from the patient who received immunosuppressive therapies. To enhance the sensitivity of detection, we then pre-amplified the DNA templates and applied EBV-BW PCR. The p-EBV-BW PCR significantly enhanced the sensitivity of EBV detection and the additional 2 patient samples were positive for EBV.

Because 10 ng of DNA is detectable in our standard agarose gel electrophoresis and the p-EBV-BW PCR method amplifies DNA 2.3 × 10^11^ times in theory, the presence of 4.3 × 10^−20^ g of DNA (about 40 base pairs) in the template is ideally detectable. Considering that the length of the BamHI W region is 125 base pairs and EBV genome contains multiple copies of BamHI W, we speculate that the presence of even 1 EBV particle in the patient cancer sample can be theoretically detectable by the p-EBV-BW PCR method. Lymphoma were formed by the patient CRC samples positive for EBV-BW-PCR and/or p-EBV-BW-PCR, and no lymphoma was formed by the patient samples negative for p-EBV-BW-PCR. Although further studies are required, these results suggest that the relative amount of EBV and/or the presence of the EBV virus particle itself in the patient sample are an important factor(s) for lymphomagenesis after xenotransplantation.

In summary, the amount of EBV in the patient cancer tissues significantly differs depending on the patients and the relative amount of EBV in the patient sample is one of the important factors that affect lymphomagenesis after xenotransplantation. To our knowledge, p-EBV-BW PCR is the first method to evaluate the risk of lymphomagenesis before xenotransplantation to prevent the loss of patient samples. Considering that no lymphoma was formed after transplantation of the patient samples negative for p-EBV-BW PCR and all lymphoma was formed from the samples positive for EBV-BW PCR/p-EBV-BW PCR, we propose that additional strategies, such as the depletion of lymphocytes from the patient samples, will be better to be performed before xenotransplantation when the patient cancer samples are positive for p-EBV-BW PCR.

## MATERIALS AND METHODS

### Patient samples

Primary human CRC tissues were obtained from the patients who admitted Kobe University Hospital. The written informed consent was obtained from each patient. The cancer type, histological grade and clinical stage (TNM classification, 7th edition) were obtained from clinical and histopathological reports. The investigation was preapproved by the Institutional Review Boards at Kobe University (permission number: 1299).

### Xenografts

All animal experiments were performed under the approval of the Kobe University Animal Care and Use Committee (permission number: 150802). All surgical procedures were performed under isoflurane anesthesia with care to minimize suffering of mice. The tissues obtained from CRC patients were minced and suspended in Matrigel (BD Biosciences), and then subcutaneously transplanted on the flank of female NOD/SCID or NSG mice (Oriental Kobo, Japan) as previously described [29, 30]. Nine PDX lines were established with the method.When the tumor reached approximately 1–2 cm in size, the tumors were harvested for analyses.

### Histological examination

A portion of the patient CRC tissues were formalin fixed for the pathological evaluation. The PDX tissues were fixed in 4% paraformaldehyde/phosphate buffered saline (PBS) for 8 hours at 4°C. The paraffin-embedded specimens were cut and stained with H.E.. Immunostaining of CD3 or CD20 was performed by an autostainer (HX System Benchmark, Ventana Medical Systems, Tucson, AZ). Primary antibodies used were a mouse monoclonal antibody for human CD3 (1:50, Dako Cytomation, Glostrup, Denmark) and a mouse monoclonal antibody for human CD20 (1:50, Dako Cytomation). The *in situ* hybridization of EBER was also conducted by the autostainer according to the manufacturer's instructions (HX System Benchmark, Ventana Medical Systems). A specific probe for EBER was obtained from Ventana Medical Systems.

### Flow cytometry analysis

PDX tissues were dissociated as previously described [31]. Briefly, the tumor tissues were dissociated with collagenase III (Worthington Biochemical Corporation) and were blocked with normal mouse IgG (1:100, Wako). The cells were stained with FITC-conjugated anti-EpCAM (1:10, Biolegend), and PE-Cy7-conjugated anti-human CD45 (1:100, BD) antibodies. Mouse cells and dead cells were depleted by using anti-mouse H-2Kd (1:40, eBioscience) and biotin-conjugated anti-CD45 (1:40, BD) antibodies and PE-Cy-5 streptavidin, and 4′,6-diamidino-2-phenylindole, respectively. The profile was analyzed using a FACS Aria III cell sorter (BD Biosciences).

### IgH gene rearrangement assay

DNA was isolated from a portion of the patient primary CRC and the PDX tissues using DNeasy Blood & Tissue Kit (QIAGEN) according to the manufacturer's instructions. Clonality of the lymphocytes within the PDXs was analyzed by PCR-based IgH gene rearrangement assay using the primers as previously described [7]. PCR that amplifies the IgH variable region was performed with KOD DNA polymerase Mix (TOYOBO). Amplification fragment were visualized with ethidium bromide agarose gel electrophoresis. Clonality was determined by the presence of single and unique sized band. PCR of glyceraldehyde-3-phosphate dehydrogenase (GAPDH) was performed as a control using the following GAPDH primers: forward 5′;-AGAAGGCTGGGGCTCATTTG-3′, and reverse 5′-AGGGGCCATCCACAGTCTTC-3′.

### Pre-amplification and PCR-based assay of the EBV-BamHI W region

EBV BamHI W region was amplified by PCR using the 60 ng genomic DNA which was extracted from the patient primary CRC or the PDX tissue as a template. Primers used were as follows: forward 5′-CGGTCGCCCAGTCCTACCAG-3′, and reverse 5′-CCTGGAGAGGTCAGGTTACT-3′. Pre-amplification was performed using the same primer sets when necessary. Pre-amplified sample was diluted 8 times before the PCR amplification of the EBV BamHI W region. The protocol for the pre-amplification was follows: at 98°C for 2 minutes, and then followed by 15 cycles of 98°C for 2 minutes, 56°C for 10 seconds and 68°C for 30 seconds. PCR was performed with KOD DNA polymerase Mix as follows: at 98°C for 2 minutes, and then followed by 30 cycles of 98°C for 10 seconds, 53°C for 10 seconds and 68°C for 20 seconds. Amplified fragments were visualized with ethidium bromide agarose gel electrophoresis.

### Semi-quantitative reverse transcription PCR

RNA was extracted from the patient CRC and PDX tissues using Trizol Reagent (Invitrogen) according to the manufacturer's instructions. After the reverse transcription (RT) using MultiScrib Reverse Transcriptase (Applied Biosystems), PCR were performed using primer sets as follows: EBNA1 forward 5′-GGATGCGATTAAGGACCTTGTT-3′, and reverse 5′-CGTCAAAGCTGCACACAGTC-3′; EBNA2 forward 5′-CTACTCACGGTACTACAAAGGC-3′, and reverse 5′-CCGTGGTTCTGGACTATCTGGA-3′; EBNA3C forward 5′-CAAGGTGCATTTACCCCACTG-3′, and reverse 5′-GGGCAGGTCCGTGAGAACT-3′; LMP1 forward 5′- CCTTGGTCTACTCCTACTGATG-3′, and reverse 5′- TTACCAAGTAAGCAGCCAAAGATG-3′; GAPDH forward 5′-AGAAGGCTGGGGCTCATTTG-3′, and reverse 5′-AGGGGCCATCCACAGTCTTC-3′. PCR was performed as follows: at 98°C for 2 minutes, and then followed by 40 cycles of 98°C for 10 seconds, 52°C for 10 seconds and 68°C for 20 seconds. Data were normalized to the amount of GAPDH expression.

Expression of EBV miRNAs, BART 1-5p and BART 7 were analyzed by semi-quantitative RT- PCR as previously described [32]. The primers were as follows: BART 1-5p RT 5′-CTCAACTGGTGTCGTGGAGTCGGCATTCAGTTGAGACAGCACG-3′, forward 5′-ACACTCCAGCTGGGTCTTAGTGGAAGTGAC-3′, and probe 5′-TTCAGTTGAGACAGCACG-3′; BART 7 RT 5′-CTCAACTGGTGTCGTGGAGTCGGCAATTCAGTTGAGCCCTGGAC-3′, forward 5′-ACACTCCAGCTGGGCATCATAGTCCAGTGT-3′, and probe 5′-TTCAGTTGAG CCCTGGAC-3′; universal reverse 5′-CAACTGGTGTCGTGGAGTCGGCAA-3′. Real-time PCR was performed in the Thermal Cycler Dice (TAKARA), as follows: at 50°C for 2 minutes and 98 °C for 10 minutes, and then followed by 40 cycles of 95°C for 15 seconds and 60°C for 1 minute. Data were normalized by the amount of small nuclear RNA expression, SNORD 48.

## SUPPLEMENTARY MATERIALS FIGURES


