# Supplementary material for: Evaluation of the risk of lymphomagenesis in xenografts by the PCR-based detection of EBV BamHI W region in patient cancer specimens
Source: Oncotarget. 2016 Jun 29;7(31):50150–60. doi: 10.18632/oncotarget.10322 (PMC5226574; doi:10.18632/oncotarget.10322)
Supplement: Supplementary file 1 [file oncotarget-07-50150-s001.pdf]

Evaluation of the risk of lymphomagenesis in xenografts by the PCR-based detection of EBV BamHI W region in patient cancer specimens

SUPPLEMENTARY FIGURES

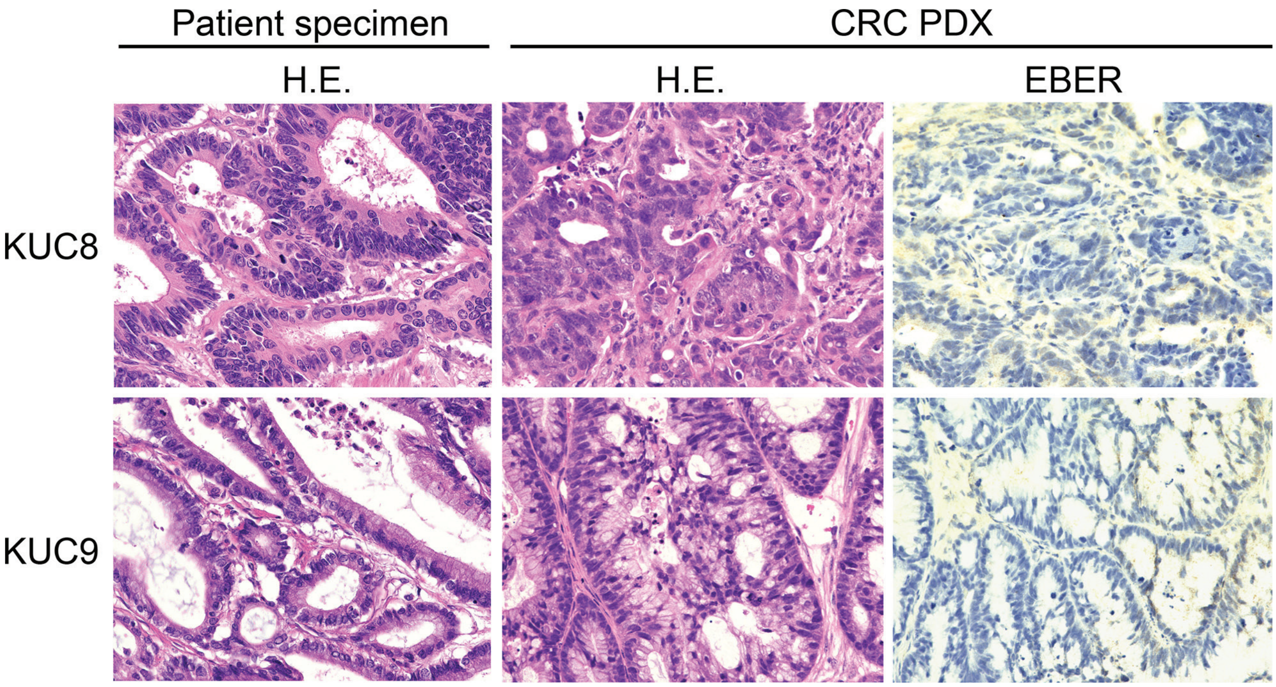

Supplementary Figure S1: Establishment of the CRC PDXs from human CRC specimens. Representative sections of the patient specimens and CRC PDXs (H.E., left and middle panels) (original magnification, ×200). EBER was undetectable by EBER-ISH in CRC PDXs (right panels).

A

		EBNA1	EBNA2	EBNA3C
Patient CRC	KUC4	+	+	+
	KUC11	-	-	-
DLBCL PDX	KUC4	+	+	+
	KUC11	+	+	+

B

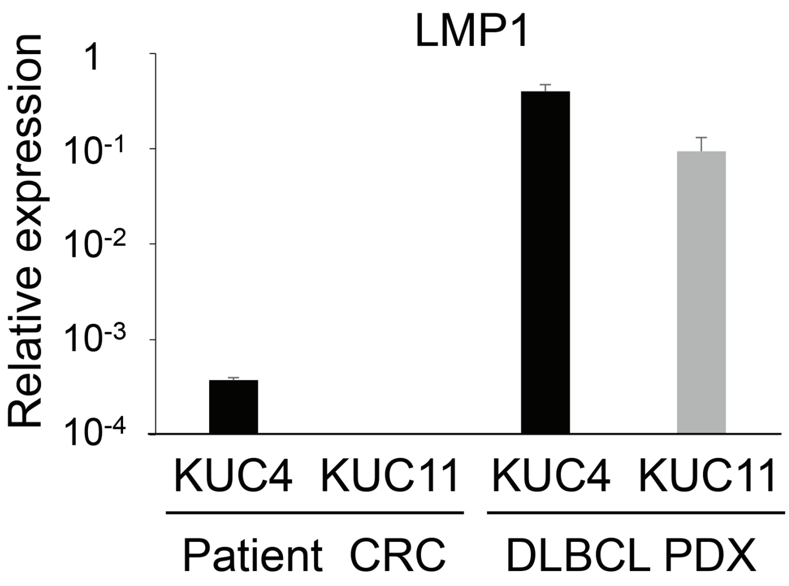

Supplementary Figure S2: Expression levels of the EBNAs and LMP1 in the patient CRC specimens and DLBCL PDXs. **A.** Expression of EBNA mRNA in the DLBCL PDXs and corresponding patient CRC specimens were analyzed by semi-quantitative RT-PCR. GAPDH was amplified as an internal control. Expression of mRNA is presented as undetectable (-) or detectable (+). **B.** Expression levels of LMP1 in the DLBCL PDXs and the corresponding patient CRC specimens were analyzed by semi-quantitative RT-PCR. Amount of the expression level of GAPDH was set as 1. The data are mean + S.D. (n=2).

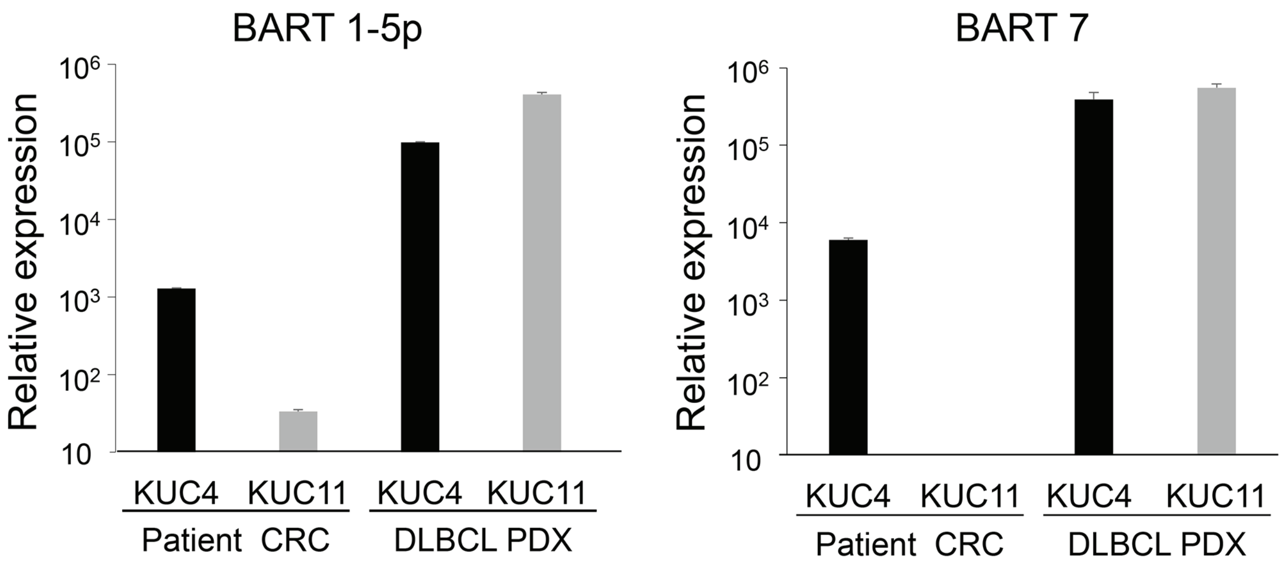

Supplementary Figure S3: Expression levels of the BART miRNAs in the patient CRC specimens and DLBCL PDXs. Expression levels of BART 1-5p and BART 7 mRNA in the DLBCL PDXs and the corresponding patient CRC specimens were analyzed by semi-quantitative RT-PCR. Amount of the expression level of an internal control small RNA, SNORD 48, was set as 1. The data are mean + S.D. (n=2).
